# Kyoto Classification-Based Predictive Factors Associated with the Development of Gastric Cancer After *Helicobacter pylori* Eradication: A Prospective Multicenter Observational Study

**DOI:** 10.3390/diagnostics15182376

**Published:** 2025-09-18

**Authors:** Shun Takayama, Osamu Dohi, Ryusuke Horie, Takeshi Yasuda, Tomoko Ochiai, Naoto Iwai, Eiko Imamoto, Tomohisa Takagi, Osamu Handa, Hideyuki Konishi, Takashi Ando, Yuji Naito, Toshiki Takemura, Yoshito Itoh

**Affiliations:** 1 Molecular Gastroenterology and Hepatology, Graduate School of Medical Science, Kyoto Prefectural University of Medicine, Kyoto 6028566, Japan; 2 Department of Gastroenterology, Japan Community Health Care Organization Kyoto Kuramaguchi Medical Center, Kyoto 6038151, Japan; 3 Department of Gastroenterology, Akashi City Hospital, Akashi 6738501, Japan; 4 Division of Gastroenterology, Department of Internal Medicine, Kawasaki Medical School, Kurashiki 7010192, Japan; 5 Ando Clinic, Kyoto 6038125, Japan; 6 Department of Human Immunology and Nutrition Science, Graduate School of Medical Science, Kyoto Prefectural University of Medicine, Kyoto 6028566, Japan; 7 Department of Gastroenterology, Gakken-toshi Hospital, Kyoto 6190238, Japan; 8 Takemura Clinic, Kyotanabe 6100334, Japan

**Keywords:** gastric cancer, *Helicobacter pylori*, Kyoto Classification of Gastritis, map-like redness, regular arrangement of collecting venule

## Abstract

**Background/Objectives**: This study aimed to identify specific endoscopic findings associated with the development of GC following successful *H. pylori* eradication. **Methods**: This prospective multicenter observational study included patients who underwent annual surveillance endoscopy after successful *H. pylori* eradication therapy between September 2013 and June 2019. Endoscopic findings were evaluated one year after eradication therapy and analyzed using the Kyoto Classification of Gastritis to identify factors associated with GC development. **Results**: A total of 465 patients were included, including 49 patients with GC and 416 patients without GC. At the initial endoscopic assessment (median, 0.96 years post-eradication), emergence of map-like redness and invisible regular arrangement of collecting venule (RAC) as independent predictors of GC (map-like redness: hazard ratio [HR], 2.561; 95% confidence interval [CI], 1.362–4.572; *p* = 0.003; invisible RAC: HR, 3.131; 95% CI, 1.078–9.091; *p* = 0.036). Patients with map-like redness or invisible RAC showed a significantly higher incidence of GC than those without map-like redness or invisible RAC (*p* < 0.001 and *p* < 0.001, respectively). Notably, map-like redness and visible RAC appeared in 13% and 28.4% of cases within the first year after eradication, respectively. **Conclusions**: Map-like redness and invisible RAC were identified as independent predictors of GC following *H. pylori* eradication and may serve as early predictive indicators, appearing within one year of successful eradication. This finding underscores the importance of early surveillance endoscopy in identifying patients at elevated risk for GC.

## 1. Introduction

Gastric cancer (GC) is the fifth most common cancer in the world [1] and one of the most common cancers in Japan [2]. *Helicobacter pylori* (*H. pylori*) is widely known as a risk factor for GC [3,4]. It has been reported that the eradication of *H. pylori* reduces the risk of GC in patients with *H. pylori* infection [5,6]. In Japan, eradication therapy for *H. pylori*-positive gastritis is already being widely administered for the prevention of GC. However, even if the eradication of *H. pylori* is successful, the risk of developing GC remains [7,8]. Hence, surveillance endoscopy is necessary to detect GC after the eradication of *H. pylori* [9].

Recently, the Kyoto Classification of Gastritis was proposed to evaluate the risk factors for GC based on endoscopic findings of gastritis, including *H. pylori* infection status [10]. Previous retrospective studies have shown that gastric mucosal atrophy, intestinal metaplasia, map-like redness, and invisible regular arrangement of collecting venules (RAC) detected after successful eradication therapy are associated with an increased risk of GC [11,12,13,14,15]. There are only a few prospective studies on the risk factors for GC in the long-term course after *H. pylori* eradication [16,17]. Severe gastric atrophy, male sex, and a history of multiple gastric cancers are key risk factors for metachronous gastric cancer after *H. pylori* eradication during long-term endoscopic surveillance. However, risk assessment based on the Kyoto classification has not been sufficiently evaluated in these reports.

Most GCs after *H. pylori* eradication are detected within a relatively short period following eradication, with the median period being reported to be 36.0 to 39.0 months [18,19]. Therefore, it is important to identify patients who have high-risk factors for the development of GC following *H. pylori* eradication at an early stage according to the Kyoto Classification of Gastritis. This study aimed to prospectively identify specific endoscopic findings associated with GC following successful *H. pylori* eradication during the early phase of surveillance endoscopy.

## 2. Materials and Methods

This prospective multicenter observational study was conducted at the Kyoto Prefectural University of Medicine, Kyoto Kuramaguchi Medical Center, and Gakken–Toshi Hospital. We enrolled consecutive patients who underwent successful eradication therapy for active *H. pylori* infection between September 2013 and June 2019. Patient information, including past history (peptic ulcer, early gastric cancer), comorbidities (hypertension, dyslipidemia, and diabetes), and lifestyle habits (history of smoking and alcohol consumption), was recorded at the time of enrollment using a questionnaire. The inclusion criterion was negative results after *H. pylori* eradication therapy. Patients of <16 years of age and those with incomplete clinical information were excluded from the study. Patients with a follow-up period of <3 years were also excluded due to insufficient observation time, to minimize the potential influence of incomplete follow-up on the observed incidence of GC after *H. pylori* eradication. The enrolled patients underwent annual surveillance endoscopy for GC after *H. pylori* eradication and followed up through December 2023.

Informed consent was obtained from all participants enrolled in this study. This study was approved by the Institutional Review Board of the Kyoto Prefectural University of Medicine on 19 August 2013 (ERBE-12-4) and was conducted in accordance with the tenets of the Declaration of Helsinki. This study is a secondary analysis of a prospective observational study, originally designed to investigate the incidence of gastric cancer after successful *H. pylori* eradication, and registered with the University Hospital Medical Information Network Clinical Trials Registry (UMIN000028760). In this secondary analysis, the primary endpoint was to identify the endoscopic features associated with the development of GC after successful *H. pylori* eradication therapy. The secondary endpoint was the identification of patient characteristics, such as past history, lifestyle habits, and comorbidities, which are risk factors for GC after *H. pylori* eradication.

The *H. pylori* infection status was determined using at least one of the following clinical tests: rapid urease test (PyloriTek; Serim Research Corp., Elkhart, IN, USA) using two pieces of gastric mucosa, anti-*H. pylori* IgG serological test (cutoff value: 10 U/mL), 13C-urea breath test (Otsuka, Tokyo, Japan), histological examination, or stool antigen test. Patients with active *H. pylori* infection received *H. pylori* eradication therapy, which consisted of administration of amoxicillin, clarithromycin, and a proton pump inhibitor or potassium-competitive acid blocker twice daily for one week. If the initial eradication therapy was unsuccessful, a second regimen consisting of amoxicillin, a proton pump inhibitor or potassium-competitive acid blocker, and metronidazole, instead of clarithromycin, was administered. Successful *H. pylori* eradication was confirmed using at least one of the following reliable clinical tests: 13C urea breath test (UBit; Otsuka, Tokyo, Japan) and stool antigen test.

Endoscopic findings of the background mucosa were evaluated using a GIF 240 series, GIF 260 series, or GIF 290 series endoscope (Olympus, Tokyo, Japan), or an EG-L590ZW, EG-600WR, or EG-L600ZW endoscope with the LASEREO 4450 endoscopic system (Fujifilm Co., Tokyo, Japan) by each endoscopist in each institution. Endoscopic images of the stomach, including antegrade and retroflex views of each part of the stomach, were obtained at least 22 times according to the systematic stomach screening protocol [20] using white-light imaging (WLI), narrow-band imaging (NBI), blue laser/light imaging (BLI), or linked color imaging (LCI).

**Figure 1:**
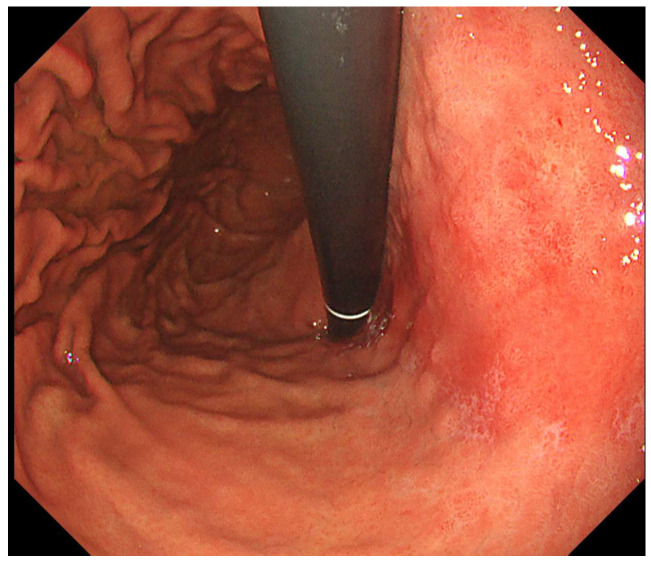
Map-like redness.

**Figure 2:**
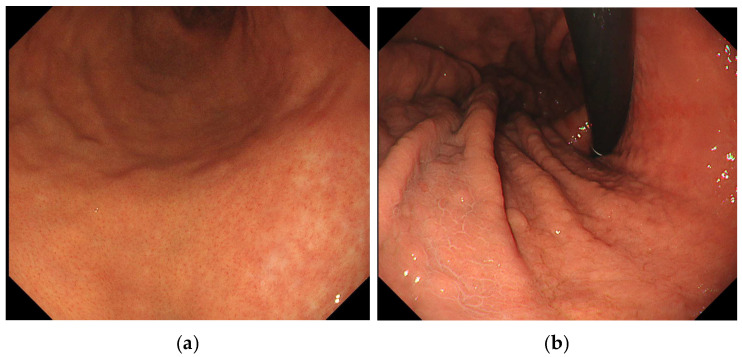
(**a**) RAC, (**b**) invisible RAC. RAC, regular arrangement of collecting venules.

We used WLI to evaluate endoscopic findings according to the Kyoto Classification of Gastritis [10] as follows: mucosal atrophy, intestinal metaplasia, enlarged folds, nodularity, diffuse redness, foveolar hyperplastic polyp, xanthoma, patchy redness, map-like redness, and RAC in the remaining fundic gland region. In this study, atrophy was defined as A0 (C0, C1), A1 (C2, O3), and A2 (O1, O2, O3) according to the Kimura–Takemoto classification [21]. Intestinal metaplasia was defined as IM0 (none), IM1 (antrum), and IM2 (antrum and corpus) according to multiple, ashen, nodular, cobblestone-like lesions typically observed in the atrophic mucosa. An enlarged fold was defined as a fold of more than 5 mm diameter under insufflation of the stomach [22]. Nodularity was defined as multiple small miliary vesicles mainly located in the gastric antrum. Diffuse redness was defined as uniform redness with continuous expansion observed in non-atrophic mucosa, mainly in the corpus [23]. Foveolar hyperplastic polyp was defined as a reddish polyp with a regular, large mucosal pattern and microvascular dilation. A xanthoma was defined as a yellowish-white and well-demarcated patchy area. Patchy redness was defined as localized reddish macules measuring 10 mm. Map-like redness was defined as multiple flat or slightly depressed erythematous lesions of various shapes, each more than 10 mm in size (Figure 1). RAC was defined as starfish-like red spots arranged regularly and visible through the non-atrophic mucosal surface of the fundic gland region (Figure 2) [24]. Invisible RAC was defined as the absence of clearly visible RAC in the non-atrophic mucosa of the fundic gland area.

Each finding was evaluated during annual esophagogastroduodenoscopy, starting one year after *H. pylori* eradication. The association between each endoscopic finding at one year after *H. pylori* eradication and the subsequent risk of gastric cancer development was evaluated. All endoscopic findings were evaluated by an endoscopist during the examination and confirmed by another endoscopist after examination.

GC that was newly detected during surveillance endoscopy after *H. pylori* eradication was histologically confirmed using biopsy specimens in each institution. All newly detected GC cases were treated using endoscopic or surgical resection. Diagnoses were categorized by experienced clinical pathologists at each institution. In cases of discrepancies between biopsy and resected specimens, the final diagnosis was based on the features of the resected specimen.

## 3. Statistical Analysis

The statistical significance of differences in continuous variables was determined using Student’s *t*-test. Univariate and multivariate analyses using a Cox proportional hazard regression model were performed to evaluate factors associated with the development of GC. Factors that showed a *p* value < 0.05 in the univariate analysis were entered into a multivariate analysis to identify the independent predictors of the development of GC. Statistical significance was set at *p* < 0.05. The results are expressed as hazard ratios (HRs) with 95% confidence intervals (CIs). The cumulative incidence of GC was calculated using the Kaplan–Meier method, and significance was ascertained using the log-rank test. All statistical analyses were performed using IBM SPSS (ver. 27.0; IBM SPSS, Chicago, IL, USA).

## 4. Results

**Figure 3:**
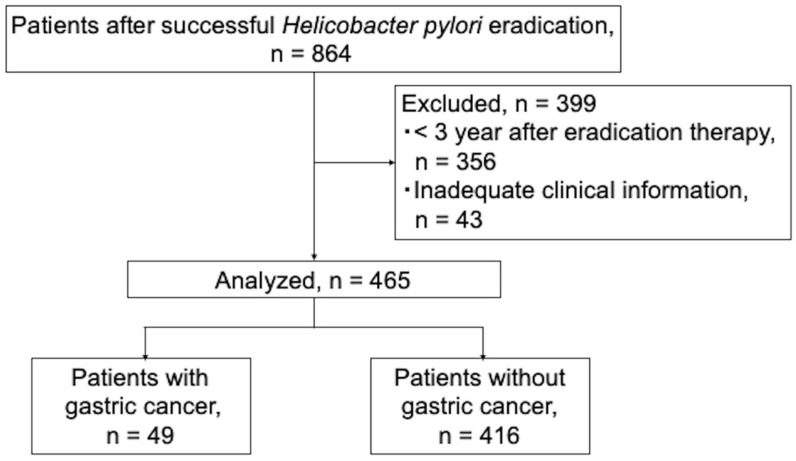
Flowchart of patient selection.

A total of 864 patients who underwent successful *H. pylori* eradication therapy were enrolled in the study. Of these, 43 patients with incomplete clinical information and 356 patients with a short follow-up period of <3 years after eradication therapy were excluded. Therefore, 465 patients were included in this study (Figure 3).

The clinical characteristics of the patients are summarized in Table 1. The study population included 252 men (54.2%) and 213 women (45.8%) with a median age of 65 years (IQR 58–71). The reasons for *H. pylori* eradication therapy were chronic active gastritis, peptic ulcer, early gastric cancer, and MALT lymphoma in 304 (65.4%), 44 (9.5%), 115(24.7%), and 2 (0.4%) patients, respectively. In terms of comorbidities, 141 patients (30.3%) had hypertension, 102 (21.9%) had dyslipidemia, and 44 (9.5%) had diabetes. The median time to first endoscopy was 0.94 year after *H. pylori* eradication therapy, and the median follow-up period was 6.06 years. Patients were divided into two groups: those with newly detected GC (cancer group) and those without GC (non-cancer group) after *H. pylori* eradication. During the follow-up period, 64 GCs were identified in 49 patients. A comparison of the clinical characteristics of the patients in the cancer and non-cancer groups is outlined in Table 1. Patients in the Cancer group were significantly more likely to be older and male than those in the non-cancer group (83.7% vs. 50.7% and 70 vs. 65 years, *p* < 0.001 and *p* < 0.001, respectively). In addition, a history of early GC (65.3% vs. 19.9%, *p* < 0.001), hypertension (49.0% vs. 25.5%, *p* = 0.002), smoking (63.3% vs. 32.7%, *p* < 0.001), and alcohol consumption (73.5% vs. 50.0%, *p* = 0.002) was significantly more likely to be observed in the cancer group than in the non-cancer group.

Table 2 compares the endoscopic features of the cancer and non-cancer groups. Based on endoscopic findings, open-type atrophy (91.8% vs. 66.1%, *p* = 0.035), intestinal metaplasia (59.2% vs. 35.1%, *p* = 0.003), xanthoma (38.8% vs. 22.3%, *p* = 0.024), map-like redness (38.8% vs. 22.3%, *p* < 0.001), and invisible RAC (93.9% vs. 63.2%, *p* < 0.001) were significantly more likely to be observed in the cancer group than in the non-cancer group.

Multivariate analysis using Cox proportional hazards modeling was performed to identify the endoscopic findings that were independently associated with the development of GC after *H. pylori* eradication. Five factors (open-type atrophy, intestinal metaplasia, xanthoma, map-like redness, and invisible RAC) with *p* values of <0.05 in univariate analyses were included in the multivariate analysis. The results showed that map-like redness (HR, 2.561; 95% CI, 1.362–4.572; *p* = 0.003) and invisible RAC (HR, 3.131; 95% CI, 1.078–9.091; *p* = 0.036) after the eradication of *H. pylori* were predictive factors for the development of GC in the initial phase (Table 3).

**Figure 4:**
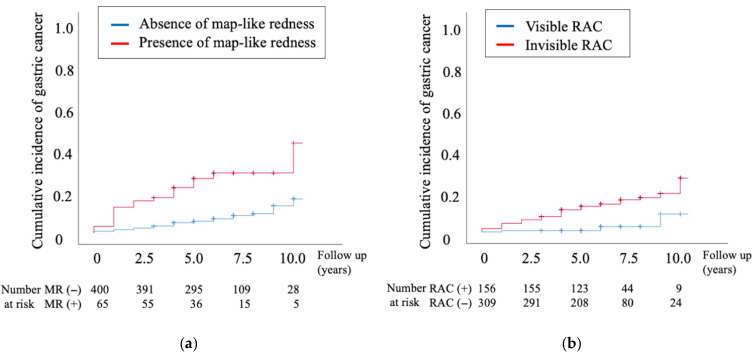
Cumulative incidence of gastric cancer according to endoscopic findings: (**a**) map-like redness; (**b**) RAC. RAC, regular arrangement of collecting venules.

Kaplan–Meier analyses were conducted to determine the cumulative incidence of GC according to the presence of map-like redness and invisible RAC, which were independent risk factors for GC in the initial phase (Figure 4). Patients with map-like redness showed a significantly higher incidence of GC than those without map-like redness (5-year and 10-year incidence rates: 26.1% vs. 6.7% and 43.0% vs. 16.3%, respectively, *p* < 0.001, Figure 4a). Patients with invisible RAC showed a significantly higher incidence of GC than those with visible RAC (5-year incidence rate, 12.4% vs. 0.6%, respectively; 10-year incidence rate, 26.0% vs. 8.7%, respectively; *p* < 0.001; Figure 4b).

**Figure 5:**
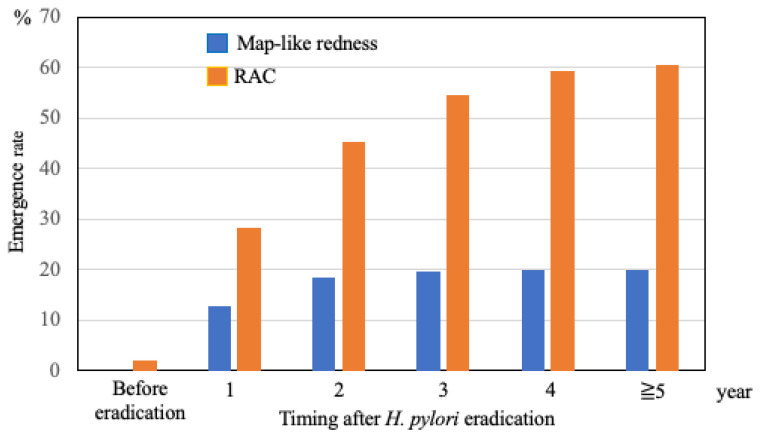
Emergence rates of map-like redness and RAC after *H. pylori* eradication. *H. pylori*, Helicobacter pylori; RAC, regular arrangement of collecting venules.

The emergence rates of map-like redness and visible RAC were evaluated after *H. pylori* eradication (Figure 5). Map-like redness was not observed in any of the patients before *H. pylori* eradication. However, it was noted in 13% of cases within the first year after eradication and in 20% in the fourth year, and its incidence remained almost unchanged over the years after eradication. RAC was observed in only 9 cases before *H. pylori* eradication. However, it was detected in 28.4% of the patients within one year of eradication, and its incidence gradually increased over time, reaching 60.4% after 5 years.

The demographic characteristics and clinicopathological findings of 49 patients with 64 GCs during the follow-up period are shown in Table 4. The median time from *H. pylori* eradication to detection of GC detection was 3.8 years. The median size of GCs was 8.0 mm. Regarding the macroscopic type, GCs were elevated in 12 cases, flat in 8, and depressed in 44. Regarding histological type, the GCs included 59 well-differentiated, four moderately differentiated, and one poorly differentiated adenocarcinoma. Among a total of 64 GCs, 60 intramucosal and 4 submucosal cancers, including 3 superficial and 1 deep submucosal cancer, were treated using endoscopic submucosal dissection (ESD). One patient who underwent surgery showed superficial submucosal invasion with lymph node metastasis.

## 5. Discussion

To the best of our knowledge, this is the first prospective cohort study to investigate the correlation between endoscopic findings (according to the Kyoto Classification of Gastritis) and GC detection after successful *H. pylori* eradication therapy. Our results showed that the detection of map-like redness and invisible RAC in the first endoscopy within a median of one year after *H. pylori* eradication were independent predictors of the detection of GC after *H. pylori* eradication. Therefore, these findings emphasize the importance of early surveillance endoscopy in identifying patients at elevated risk for GC.

It has been previously reported that atrophy and intestinal metaplasia are risk factors for GC after *H. pylori* eradication [8,24]. Recently, Kawamura et al. reported that open-type atrophy, intestinal metaplasia of more than 30% in the corpus, invisible RAC in the angulus, and map-like redness in the corpus were significantly high-risk factors for GC, regardless of the *H. pylori* infection status [15]. On the other hand, Shibukawa et al. reported that gastric xanthoma was a predictive marker for early GC detected after *H. pylori* eradication [25]. However, these retrospective cohort studies showed that the period of post-*H. pylori* eradication rate was not uniform during the evaluation of endoscopic findings. Therefore, our study has the advantage of prospectively evaluating the endoscopic findings after *H. pylori* eradication.

Map-like redness is characterized by irregular, patchy areas of redness in the gastric mucosa. Histologically, this feature is closely associated with intestinal metaplasia, a precancerous condition of the gastric mucosa [26]. Map-like redness is a specific endoscopic finding that appears in 21–51% of patients after *H. pylori* eradication [14,27,28] and is also a risk factor for GC after *H. pylori* eradication [13,14,15]. Interestingly, we found that the emergence of map-like redness became apparent in approximately 13% of patients within one year and did not increase beyond 5 years after *H. pylori* eradication therapy. Therefore, map-like redness could be a predictor of high-risk GC in the early phase after *H. pylori* eradication.

In *H. pylori*-active gastritis, invisible RAC is histologically related to inflammatory changes with neutrophilic infiltration from the glandular neck zone to the foveolar epithelium in *H. pylori*-active gastritis. RAC became visible because of the rapid disappearance of neutrophilic infiltration after *H. pylori* eradication. However, mononuclear cell infiltration sometimes persists after *H. pylori* eradication. Invisible RAC (regular arrangement of collecting venules) findings have been associated with mononuclear cell infiltration. This suggests that several years may be required after eradication therapy before RAC can be visualized. Therefore, patients with visible RAC in the early phase after *H. pylori* eradication may have a reduced risk of GC owing to the disappearance of inflammation with mononuclear cell infiltration.

Of the 64 GCs detected using surveillance endoscopy in the present study, approximately 94% were intramucosal cancers, and the remaining 6% were submucosal cancers. These results suggest that annual endoscopic surveillance after *H. pylori* eradication can detect early GCs that may be treated with curative endoscopic resection. In the Kyoto global consensus report on *H. pylori* gastritis, endoscopic surveillance after *H. pylori* eradication is recommended depending on the extent and severity of atrophy [9]. Currently, there is no established guideline for the appropriate surveillance interval after *H. pylori* eradication. Given the observational nature of this study, annual surveillance endoscopy may be considered for patients with map-like redness or invisible RAC following *H. pylori* eradication, although further prospective studies are warranted to validate this approach.

Among the 465 patients enrolled in the study, 49 (10.5%) developed gastric cancer. This incidence is notably higher than those reported in previous studies [3,29] and may be partially attributed to advances in endoscopic technology over the past two decades. In particular, the introduction of high-resolution endoscopy and image-enhanced endoscopy, including NBI, BLI, and LCI, has markedly improved the detection of GC, especially flat or subtle lesions that might have been missed using conventional WLI [30,31,32,33]. These technological advancements may have contributed to the increased diagnostic yield observed in our cohort.

This study has some limitations. First, a histological evaluation of each endoscopic finding was not performed. Therefore, endoscopic evaluation is subjective and depends on the endoscopist. Second, the endoscopic findings in most cases were evaluated using WLI. It has been reported that intestinal metaplasia can be clearly observed using NBI, BLI, and LCI [34,35,36], whereas LCI can visualize map-like redness better than other modalities [37]. Therefore, there is a possibility that WLI was not sufficient for the detection of these findings. Third, diffuse-type GC was not detected in this study; therefore, it was impossible to assess its risk factors. Fourth, inter- and intra-observer agreement in the assessment of each endoscopic finding was not evaluated, which may limit the reproducibility and reliability of the results.

## 6. Conclusions

This study demonstrated that map-like redness and invisible RAC are independent predictors of GC following *H. pylori* eradication. These indicators may appear within one year of successful eradication and serve as early signs of GC risk. This finding underscores the importance of early surveillance endoscopy in identifying patients at elevated risk for GC.

## Figures and Tables

**Table 1:** Clinical characteristics of the patients. IQR, interquartile range.

Variables		Overall, *n* = 465	Cancer, *n* = 49	Non-Cancer, *n* = 416	*p* Value, Cancer vs. Non-Cancer
Sex, *n* (%)					
	Male	252 (54.2)	41 (83.7)	211 (50.7)	<0.001
	Female	213 (45.8)	8 (16.3)	205 (49.3)	
Age of eradication, median, years (IQR)		65 (58–71)	70 (66–74)	65 (57–70)	<0.001
Body mass index, median, kg/m^2^ (IQR)		22.5 (20.3–24.6)	23.3 (20.5–25.0)	22.4 (20.3–24.6)	0.440
Past history, *n* (%)					<0.001
	Chronic gastritis	304 (65.4)	15 (30.6)	289 (69.5)	
	Peptic ulcer	44 (9.5)	2 (4.1)	42 (10.1)	
	Early gastric cancer	115 (24.7)	32 (65.3)	83 (19.9)	
	MALT lymphoma	2 (0.4)	0	2 (0.5)	
Comorbidity, *n* (%)					
	Hypertension	141 (30.3)	24 (49.0)	106 (25.5)	0.002
	Dyslipidemia	102 (21.9)	16 (32.7)	98 (23.6)	0.259
	Diabetes	44 (9.5)	8 (16.3)	34 (8.2)	0.119
Smoking history, *n* (%)					<0.001
	None	299 (64.3)	18 (36.7)	280 (67.3)	
	Current/former	166 (35.7)	31 (63.3)	136 (32.7)	
Drinking history, *n* (%)					0.002
	None	222 (47.7)	13 (26.5)	208 (50.0)	
	Current/former	243 (52.3)	36 (73.5)	208 (50.0)	
Proton pump inhibitor, *n* (%)		48 (10.3)	7 (14.3)	41 (9.9)	0.510
Time to first endoscopy, median, year (IQR)		0.94 (0.74–1.17)	0.88 (0.74–1.10)	0.97 (0.75–1.23)	0.320
Follow-up period, median, year (IQR)		6.06 (4.53–7.72)	7.82 (5.73–8.59)	6.02 (4.38–7.51)	<0.001

**Table 2:** Endoscopic features at first endoscopy related to the development of gastric cancer after *Helicobacter pylori* eradication. RAC, regular arrangement of collecting venules.

Endoscopic Findings	Cancer, *n* = 49	Non-Cancer, *n* = 416	*p* Value
Atrophy, A2, *n* (%)	45 (91.8)	275 (66.1)	0.035
Intestinal metaplasia, IM2, *n* (%)	29 (59.2)	146 (35.1)	0.003
Enlarged fold, *n* (%)	3 (6.1)	19 (4.6)	0.925
Nodularity, *n* (%)	0 (0)	15 (3.6)	0.346
Diffuse redness, D2, *n* (%)	3 (6.1)	29 (7.0)	1
Foveolar hyperplastic polyp, *n* (%)	5 (4.9)	22 (5.3)	0.307
Patchy redness, *n* (%)	24 (49.0)	161 (38.7)	0.270
Xanthoma, *n* (%)	19 (38.8)	93 (22.3)	0.024
Map-like redness, *n* (%)	18 (36.7)	47 (11.3)	<0.001
Invisible RAC, *n* (%)	46 (93.9)	263 (63.2)	<0.001

**Table 3:** Multivariate analysis of endoscopic features associated with the development of gastric cancer after *H. pylori* eradication. *H. pylori*, Helicobacter pylori, CI, confidence interval; HR, hazard ratio; RAC, regular arrangement of collecting venules.

Variables	HR	95% CI	*p* Value
Atrophy, A2	2.842	0.978–8.259	0.055
Intestinal metaplasia, IM2	1.621	0.889–2.955	0.115
Xanthoma	1.548	0.867–2.765	0.140
Map-like redness	2.561	1.362–4.572	0.003
Invisible RAC	3.131	1.078–9.091	0.036

**Table 4:** Demographic characteristics and clinicopathological findings of 32 patients with 34 gastric cancers. IQR, interquartile range; ESD, endoscopic submucosal dissection.

Characteristics/Findings		49 Patients with 64 Gastric Cancers
Sex, *n* (%)		
	Male	41 (83.7)
	Female	8 (16.3)
Age, median, years (IQR)		70 (66–74)
Lesion size, median, mm, (IQR)		8.0 (6.0–13.8)
Macroscopic type, *n* (%)		
	Elevated	12 (18.8)
	Flat	8 (12.5)
	Depressed	44 (68.7)
Location, *n* (%)		
	Upper third	11 (17.2)
	Middle third	34 (53.1)
	Lower third	19 (29.7)
Histological type, *n* (%)		
	Well-differentiated adenocarcinoma	59 (92.1)
	Moderately differentiated adenocarcinoma	4 (6.3)
	Poorly adenocarcinoma	1 (1.6)
Depth of invasion, *n* (%)		
	Intramucosa	60 (93.7)
	Submucosa, superficial	3 (4.7)
	Submucosa, deep	1 (1.6)
Treatment, *n* (%)		
	ESD	63 (98.4)
	Surgery	1 (1.6)
Median time to detection, year, (range)		3.8 (1.8–6.1)

## Data Availability

The patient data used to support the findings of this study are available from the corresponding author upon request. However, identified patient data are restricted by the institutional review board of Kyoto Prefectural University of Medicine.

## References

[1] Bray F., Ferlay J., Soerjomataram I., Siegel R.L., Torre L.A., Jemal A. (2018). Global cancer statistics 2018: GLOBOCAN estimates of incidence and mortality worldwide for 36 cancers in 185 countries. CA Cancer J. Clin..

[2] Hori M., Matsuda T., Shibata A., Katanoda K., Sobue T., Nishimoto H., Japan Cancer Surveillance Research Group (2015). Cancer incidence and incidence rates in Japan in 2009: A study of 32 population-based cancer registries for the Monitoring of Cancer Incidence in Japan (MCIJ) project. Jpn. J. Clin. Oncol..

[3] Uemura N., Okamoto S., Yamamoto S., Matsumura N., Yamaguchi S., Yamakido M., Taniyama K., Sasaki N., Schlemper R.J. (2001). Helicobacter pylori infection and the development of gastric cancer. N. Engl. J. Med..

[4] Herrero R., Park J.Y., Forman D. (2014). The fight against gastric cancer—The IARC Working Group report. Best Pract. Res. Clin. Gastroenterol..

[5] Ford A.C., Forman D., Hunt R.H., Yuan Y., Moayyedi P. (2014). Helicobacter pylori eradication therapy to prevent gastric cancer in healthy asymptomatic infected individuals: Systematic review and meta-analysis of randomised controlled trials. BMJ.

[6] Lee Y.C., Chiang T.H., Chou C.K., Tu Y.K., Liao W.C., Wu M.S., Graham D.Y. (2016). Association between Helicobacter pylori eradication and gastric cancer incidence: A systematic review and meta-analysis. Gastroenterology.

[7] Saka A., Yagi K., Nimura S. (2016). Endoscopic and histological features of gastric cancers after successful Helicobacter pylori eradication therapy. Gastric Cancer.

[8] Take S., Mizuno M., Ishiki K., Yoshida T., Ohara N., Yokota K., Oguma K., Okada H., Yamamoto K. (2011). The long-term risk of gastric cancer after the successful eradication of Helicobacter pylori. J. Gastroenterol..

[9] Sugano K., Tack J., Kuipers E.J., Graham D.Y., El-Omar E.M., Miura S., Haruma K., Asaka M., Uemura N., Malfertheiner P. (2015). Kyoto global consensus report on Helicobacter pylori gastritis. Gut.

[10] Kamada T., Haruma K., Inoue K., Shiotani A. (2015). Helicobacter pylori infection and endoscopic gastritis-Kyoto classification of gastritis. Nihon Shokakibyo Gakkai Zasshi.

[11] Masuyama H., Yoshitake N., Sasai T., Nakamura T., Masuyama A., Zuiki T., Kurashina K., Mieda M., Sunada K., Yamamoto H. (2015). Relationship between the degree of endoscopic atrophy of the gastric mucosa and carcinogenic risk. Digestion.

[12] Spence A.D., Cardwell C.R., McMenamin U.C., Hicks B.M., Johnston B.T., Murray L.J., Coleman H.G. (2017). Adenocarcinoma risk in gastric atrophy and intestinal metaplasia: A systematic review. BMC Gastroenterol..

[13] Moribata K., Kato J., Iguchi M., Nakachi K., Maeda Y., Shingaki N., Niwa T., Deguchi H., Inoue I., Maekita T. (2016). Endoscopic features associated with development of metachronous gastric cancer in patients who underwent endoscopic resection followed by Helicobacter pylori eradication. Dig. Endosc..

[14] Majima A., Dohi O., Takayama S., Hirose R., Inoue K., Yoshida N., Kamada K., Uchiyama K., Ishikawa T., Takagi T. (2019). Linked color imaging identifies important risk factors associated with gastric cancer after successful eradication of Helicobacter pylori. Gastrointest. Endosc..

[15] Kawamura M., Uedo N., Koike T., Kanesaka T., Hatta W., Ogata Y., Oikawa T., Iwai W., Yokosawa S., Honda J. (2022). Kyoto classification risk scoring system and endoscopic grading of gastric intestinal metaplasia for gastric cancer: Multicenter observation study in Japan. Dig. Endosc..

[16] Mori G., Nakajima T., Asada K., Shimazu T., Yamamichi N., Maekita T., Yokoi C., Fujishiro M., Gotoda T., Ichinose M. (2016). Incidence of and risk factors for metachronous gastric cancer after endoscopic resection and successful Helicobacter pylori eradication: Results of a large-scale, multicenter cohort study in Japan. Gastric Cancer.

[17] Kamada T., Hata J., Sugiu K., Kusunoki H., Ito M., Tanaka S., Inoue K., Kawamura Y., Chayama K., Haruma K. (2005). Clinical features of gastric cancer discovered after successful eradication of Helicobacter pylori: Results from a 9-year prospective follow-up study in Japan. Aliment. Pharmacol. Ther..

[18] Majima A., Handa O., Naito Y., Dohi O., Okayama T., Yoshida N., Kamada K., Katada K., Uchiyama K., Ishikawa T. (2017). Early-stage gastric cancer can be found in improved atrophic mucosa over time from successful Helicobacter pylori eradication. Digestion.

[19] Nakata R., Nagami Y., Hashimoto A., Sakai T., Ominami M., Fukunaga S., Otani K., Hosomi S., Tanaka F., Ohira M. (2021). Successful eradication of Helicobacter pylori could prevent metachronous gastric cancer: A propensity matching analysis. Digestion.

[20] Yao K. (2013). The endoscopic diagnosis of early gastric cancer. Ann. Gastroenterol..

[21] Kimura K., Takemoto T. (1969). An endoscopic recognition of the atrophic border and its significance in chronic gastritis. Endoscopy.

[22] Tytgat G.N. (1991). The Sydney System: Endoscopic division. Endoscopic appearances in gastritis/duodenitis. J. Gastroenterol. Hepatol..

[23] Toyoshima O., Nishizawa T., Koike K. (2020). Endoscopic Kyoto classification of Helicobacter pylori infection and gastric cancer risk diagnosis. World J. Gastroenterol..

[24] Shichijo S., Hirata Y., Niikura R., Hayakawa Y., Yamada A., Ushiku T., Fukayama M., Koike K. (2016). Histologic intestinal metaplasia and endoscopic atrophy are predictors of gastric cancer development after Helicobacter pylori eradication. Gastrointest. Endosc..

[25] Shibukawa N., Ouchi S., Wakamatsu S., Wakahara Y., Kaneko A. (2019). Gastric xanthoma is a predictive marker for early gastric cancer detected after Helicobacter pylori eradication. Intern. Med..

[26] Yoshii S., Mabe K., Watano K., Ohno M., Matsumoto M., Ono S., Kudo T., Nojima M., Kato M., Sakamoto N. (2020). Validity of endoscopic features for the diagnosis of Helicobacter pylori infection status based on the Kyoto classification of gastritis. Dig. Endosc..

[27] Kotachi T., Ito M., Boda T., Kiso M., Masuda K., Hata K., Kawamura T., Sanomura Y., Yoshihara M., Tanaka S. (2018). Clinical significance of reddish depressed lesions observed in the gastric mucosa after Helicobacter pylori eradication. Digestion.

[28] Nagata N., Shimbo T., Akiyama J., Nakashima R., Kim H.H., Yoshida T., Hoshimoto K., Uemura N. (2011). Predictability of gastric intestinal metaplasia by mottled patchy erythema seen on endoscopy. Gastroenterol. Res..

[29] Fukase K., Kato M., Kikuchi S., Inoue K., Uemura N., Okamoto S., Terao S., Amagai K., Hayashi S., Asaka M. (2008). Effect of eradication of Helicobacter pylori on incidence of metachronous gastric carcinoma after endoscopic resection of early gastric cancer: An open-label, randomised controlled trial. Lancet.

[30] Yoshida N., Doyama H., Yano T., Horimatsu T., Uedo N., Yamamoto Y., Kakushima N., Kanzaki H., Hori S., Yao K. (2021). Early gastric cancer detection in high-risk patients: A multicentre randomised controlled trial on the effect of second-generation narrow band imaging. Gut.

[31] Kadota T., Abe S., Uedo N., Doyama H., Furue Y., Muto M., Nonaka S., Takamaru H., Murano T., Nakajo K. (2024). Comparison of Effective Imaging Modalities for Detecting Gastric Neoplasms: A Randomized 3-Arm Phase II Trial. Am. J. Gastroenterol..

[32] Dohi O., Yagi N., Naito Y., Fukui A., Gen Y., Iwai N., Ueda T., Yoshida N., Kamada K., Uchiyama K. (2019). Blue laser imaging-bright improves the real-time detection rate of early gastric cancer: A randomized controlled study. Gastrointest. Endosc..

[33] Ono S., Kawada K., Dohi O., Kitamura S., Koike T., Hori S., Kanzaki H., Murao T., Yagi N., Sasaki F. (2021). Linked Color Imaging Focused on Neoplasm Detection in the Upper Gastrointestinal Tract: A Randomized Trial. Ann. Intern. Med..

[34] Nomura S., Ida K., Terao S., Adachi K., Kato T., Watanabe H., Shimbo T., Research Group for Establishment of Endoscopic Diagnosis of Chronic Gastritis (2014). Endoscopic diagnosis of gastric mucosal atrophy: Multicenter prospective study. Dig. Endosc..

[35] Dohi O., Majima A., Naito Y., Yoshida T., Ishida T., Azuma Y., Kitae H., Matsumura S., Mizuno N., Yoshida N. (2020). Can image-enhanced endoscopy improve the diagnosis of Kyoto classification of gastritis in the clinical setting?. Dig. Endosc..

[36] Ono S., Kato M., Tsuda M., Miyamoto S., Abiko S., Shimizu Y., Sakamoto N. (2018). Lavender color in linked color imaging enables noninvasive detection of gastric intestinal metaplasia. Digestion.

[37] Takeda T., Asaoka D., Nojiri S., Nishiyama M., Ikeda A., Yatagai N., Ishizuka K., Hiromoto T., Okubo S., Suzuki M. (2020). Linked color imaging and the Kyoto classification of gastritis: Evaluation of visibility and inter-rater reliability. Digestion.

